# Identification of a novel ATM inhibitor with cancer cell specific radiosensitization activity

**DOI:** 10.18632/oncotarget.18034

**Published:** 2017-05-19

**Authors:** Amy J.C. Dohmen, Xiaohang Qiao, Anja Duursma, Ruud H. Wijdeven, Cor Lieftink, Floor Hageman, Ben Morris, Pasi Halonen, Conchita Vens, Michiel W.M. van den Brekel, Huib Ovaa, Jacques Neefjes, Charlotte L. Zuur

**Affiliations:** ^1^ Department of Head and Neck Oncology and Surgery, Antoni van Leeuwenhoek Hospital, Amsterdam, The Netherlands; ^2^ Division of Cell Biology, The Netherlands Cancer Institute, Amsterdam, The Netherlands; ^3^ NKI Robotics and Screening Center, The Netherlands Cancer Institute, Amsterdam, The Netherlands; ^4^ Division of Biological Stress Response, The Netherlands Cancer Institute, Amsterdam, The Netherlands; ^5^ Department of Oral and Maxillofacial Surgery, Academic Medical Center, University of Amsterdam, Amsterdam, The Netherlands; ^6^ Division of Cell Biology, The Netherlands Cancer Institute, Amsterdam, The Netherlands; ^7^ Department of Chemical Immunology, Leiden University Medical Center, Leiden, The Netherlands

**Keywords:** cancer, radiotherapy, radiosensitizer, ATM, DNA damage response

## Abstract

Treatment of advanced head and neck squamous cell carcinoma (HNSCC) is plagued by low survival and high recurrence rates, despite multimodal therapies. Presently, cisplatin or cetuximab is used in combination with radiotherapy which has resulted in minor survival benefits but increased severe toxicities relative to RT alone. This underscores the urgent need for improved tumor-specific radiosensitizers for better control with lower toxicities. In a small molecule screen targeting kinases, performed on three HNSCC cell lines, we identified GSK635416A as a novel radiosensitizer. The extent of radiosensitization by GSK635416A outperformed the radiosensitization observed with cisplatin and cetuximab in our models, while exhibiting virtually no cytotoxicity in the absence of radiation and in normal fibroblast cells. Radiation induced phosphorylation of ATM was inhibited by GSK635416A. GSK63541A increased DNA double strand breaks after radiation and GSK63541A mediated radiosensitization was lacking in ATM-mutated cells thereby further supporting the ATM inhibiting properties of GSK63541A. As a novel ATM inhibitor with highly selective radiosensitizing activity, GSK635416A holds promise as a lead in the development of drugs active in potentiating radiotherapy for HNSCC and other cancer types.

## INTRODUCTION

Of the estimated 686,000 new head and neck cancer cases per year worldwide [1], seventy percent of HNSCC patients enter the clinic with advanced stage disease and exhibit an overall 5-year survival rate of only 35–60% [2–4]. Radiotherapy (RT) serves as a backbone of first-line local therapy offered to nearly 75% of HNSCC patients. However, the success of this approach is limited on a number of fronts. First, HNSCC is associated with high rates of locoregional and distant recurrences. Second, RT is given at high doses (up to 70 Gy), which can cause considerable morbidity, such as loss of organ integrity and function (i.e. speech and swallowing). In an effort to improve cure rates and functional outcomes of locally advanced HNSCC, high-dose cisplatin chemotherapy has been integrated into the RT treatment regimens (CCRT) since the early 1980’s [5]. The concurrent CCRT regimen is thought to sensitize tumor cells to RT by virtue of obstructing repair of radiation-induced DNA breaks. However, meta-analysis of randomized trials has indicated only a moderate absolute overall survival benefit of 6.5% at 5 years for HNSCC patients upon addition of cisplatin to locoregional RT [6]. Furthermore, in addition to the high local recurrence rate in more than 50% of patients, CCRT is accompanied with a substantial increase in severe adverse events, including mucositis, dysphagia, nephrotoxicity and hematologic toxicity [7]. As an alternative to cisplatin, cetuximab —a humanized monoclonal antibody against the epidermal growth factor (EGF) receptor— has been administered before RT. To date, only one trial reported efficacy of cetuximab-RT in HNSCC [8], while a recent phase 2 randomized trial, comparing RT with concomitant cisplatin versus cetuximab, showed that cetuximab increased acute toxicity rates without a corresponding clinical benefit [9]. While CCRT is presently favoured over cetuximab-RT in routine care [10], it is clear that many HNSCC patients are not receiving benefits from the currently available treatments, highlighting an urgent need for alternatives. Among novel targeted drugs, PARP inhibitors (such as olaparib) emerged as potential radiosensitizers. Pre-clinical studies show efficient sensitization to RT in various tumor types [11–14].

Aiming to identify novel and better radiosensitizers for the treatment of HNSCC, we performed a screen to test compounds in a higher scale, with structural diversities and a broader range of targets. Compound screening allows for identification of compounds with a certain biological effect without the need for prior knowledge of the mechanism or the target, which facilitates the identification of critical targets [15]. To this end, we performed a kinase inhibitor screen on HNSCC cell lines in the absence and presence of ionizing radiation (IR). We identified GSK635416A as a novel radiosensitizer with a radiosensitization efficacy superior to that of cisplatin or cetuximab, and comparable to olaparib. Furthermore, as single agent, in the absence of IR, GSK635416A showed lower cytotoxicity compared to the other three drugs and it also did not radiosensitize normal fibroblast cells, indicating tumor-selectivity. We further characterised GSK635416A as a novel ATM inhibitor capable of impairing ATM activation following DNA damage. When used in combination with olaparib, GSK635416A induced radiosensitization was additive to olaparib induced radiosensitization, while showing no increased cytotoxicity. This combination treatment showed no increased radiosensitization or cytotoxicity in normal fibroblast cells. Taken together, our findings provide a basis to further explore new RT combination options with GSK635416A.

## RESULTS

### Identification of a novel radiosensitizing compound

**Figure 1:**
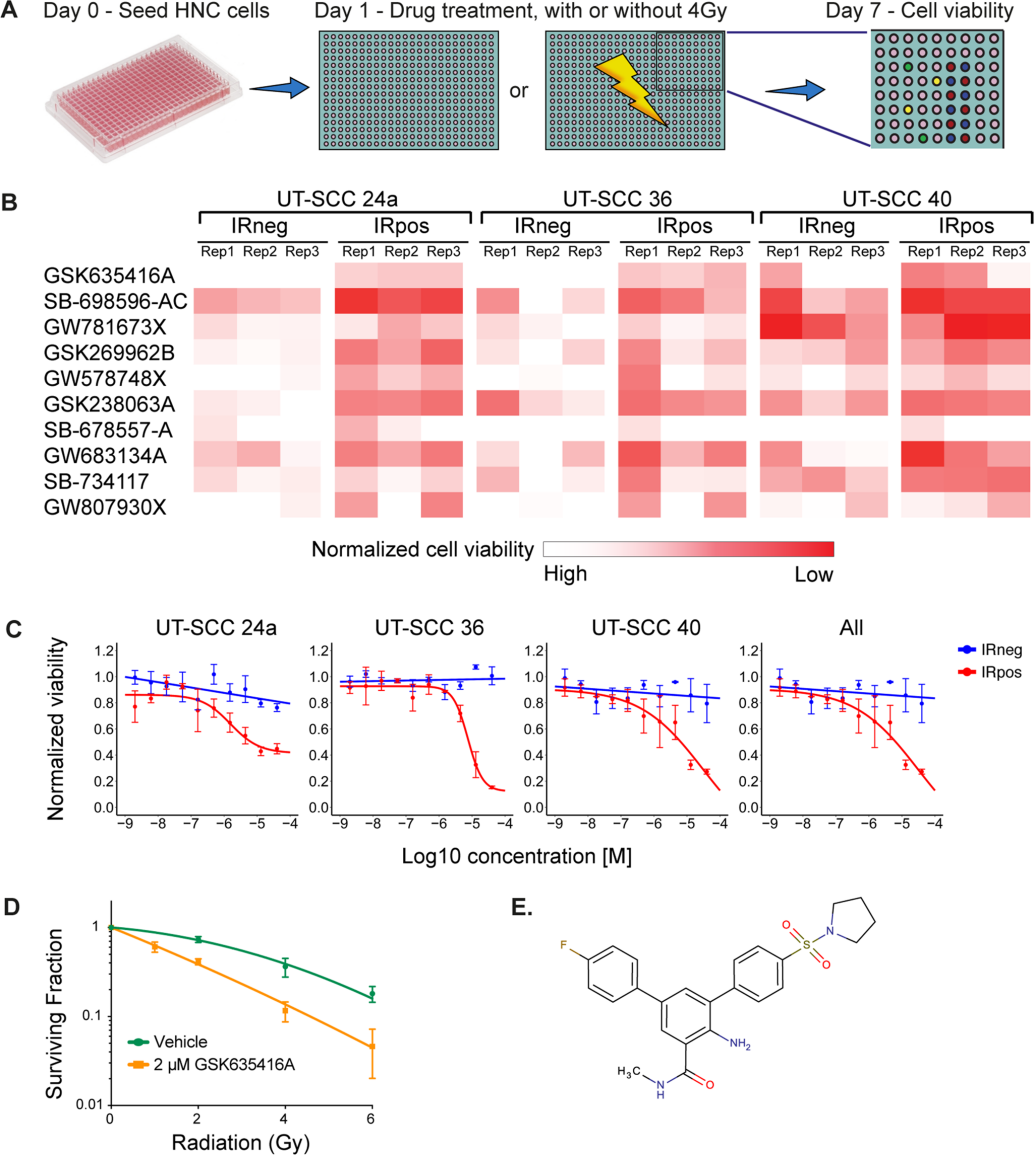
A kinase inhibitor screen identifies GSK635416A as a potential novel radiosensitizer for head and neck cancer (**A**) Schematic overview of the screening procedure. (**B**) Top 10 compounds as identified in the primary screen performed in triplicate. The heat map is a graphic representation of normalized cell viability at day 7 at a concentration of 500 nM, depicted by colour intensity. The three replicate values depict the reproducibility of the effect of the compound on cell viability in three HNSCC cell lines. IRneg is the effect on non-radiated cells; IRpos is the effect in combination with 4 Gy radiation. (**C**) Validation of top hit GSK635416A library compound. Shown are dose-response curves of GSK635416A in the absence and presence of 4 Gy radiation in UT-SCC-24a, UT-SCC-36, UT-SCC-40 HNSCC cells and a graph representing the effect on all three cell lines. The IR-effect was eliminated by normalizing to negative controls that received IR. (Data shown as mean from three independent experiments, with SEM.) (**D**) CFA on UT-SCC-36 cultured in the presence or absence of 2 μM GSK635416A and exposed to different doses of radiation. (Data shown are the mean of three independent experiments, with SD.) (**E**) Structure of GSK635416A.

To identify novel radiosensitizing compounds for HNSCC, we screened the GSK-PKIS kinase library consisting of 356 kinase inhibitors, in three HNSCC cell lines (UT-SCC-24a, −36 and −40) in the presence (IRpos) and absence (IRneg) of 4 Gy IR (Figure 1A). Cell viability was measured at day 7. Values were normalized to negative controls and IRpos values of each compound were then compared to IRneg to determine the radiosensitizing effects. A cell-viability heat-map example that visualizes the leading compound candidates at 500 nM ranked by the largest mean difference between IRneg and IRpos for the three cell lines and each replicate, is shown in Figure 1B. The *p*-values and adjusted *p*-values for these differences were all significant (< 0.00016 and < 0.00077, respectively).

Next, we validated the 17 leading candidates (the top 5 compounds in the following categories: 50 nM, 500 nM and 5 µM separately, and all concentrations taken together) over a wide concentration range. This yielded a single outstanding compound, GSK635416A, exhibiting the greatest mean difference between IRpos and IRneg for all variables (See Supplementary Table 1). The dose-response curve of GSK635416A in three cell lines (Figure 1C) showed significant IR-dependent cell kill in IRpos. However, the cytotoxicity of GSK635416A, i.e. decrease in cell viability in the absence of IR, is limited, consequently producing a large window between the two curves hence depicting the potential radiosensitization. Taken together, these results suggest that GSK635416A can act as a radiosensitizer with limited cytotoxicity. To further assay for radiosensitizing properties, we performed a colony forming assay (CFA) at 2 µM GSK635416A and various IR doses in UT-SCC-36 (Figure 1D). This concentration was chosen based on the viability assay results at which 2 µM GSK635416A showed a significant decrease in cell viability only when combined with IR. Plating efficiencies (PE) in the CFA were not different and did not decrease under 2 µM of GSK635416A treatment compared to vehicle treated controls, thereby confirming a lack of clonogenic cell death at this drug concentration without IR (Supplementary Figure 1A). The results of the CFA showed a strong radiosensitizing activity of GSK635416A with a radiation dose enhancement factor (DEF) of 1.99 (DEF_37_ 1.99, ± SD: 0.19) (Figure 1D). For comparison, a DEF_37_ of 1.90 for cisplatin in a UT-SCC cell line [16] and a DEF_37_ of 1.08–1.61 for olaparib in various cancer cell types [11, 17, 18] have been reported for similar conditions. The structure of GSK635416A is shown in Figure 1E and is unrelated to olaparib or cisplatin.

### Comparing GSK635416A to radiosensitizers currently used in HNSCC

**Figure 2:**
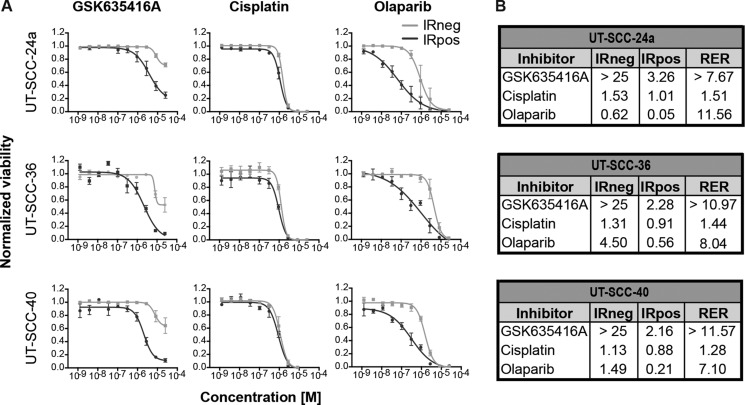
Comparison of GSK635416A to the current clinical radiosensitizers cisplatin and olaparib in various HNSCC cell lines Shown on the left (**A**) are the dose-response curves of (resynthesized) GSK635416A, cisplatin and olaparib in UT-SCC-24a, UT-SCC-36 and UT-SCC-40. The IRneg line (grey) represents the cytotoxicity effect of the compound alone. The IRpos line (black) represents the effect of the compound combined with 4 Gy radiation. The IR-effect was eliminated by normalizing to negative controls that received IR. Depicted on the right (**B**) are the corresponding calculated IC50 values (μM) for IRneg and IRpos and the determined radiation enhancement ratio (RER). (Data shown are the mean of three independent experiments, with SEM).

To compare GSK635416A to the radiosensitizers cisplatin, cetuximab and olaparib that are presently used or tested in the clinic, we generated dose-response curves using a 7-day cell viability assay. During this 7-day assay, cells were continuously exposed to the drugs. This was done since wash-out experiments revealed the highest cell kill with long drug exposures (Supplementary Figure 2). Significant differences between IRneg and IRpos data points were observed for GSK635416A and olaparib (Figure 2A), but not for cisplatin (Figure 2A) and cetuximab (Supplementary Figure 3A). This implies that cisplatin and cetuximab exhibits poor radiosensitizing effects in the three HNSCC cell lines tested. Although olaparib treatment showed a robust reduction of cell viability when combined with IR, it also resulted in cytotoxicity at higher concentrations. In contrast, the IRneg curve of GSK635416A did not reach the IC_50_ in any of the three cell lines, reported as ‘> 25 µM’ in Figure 2B, illustrating again limited cytotoxicity of GSK635416A. To quantify these observations, we calculated the radiation enhancement ratio (RER) from the reported IC_50_’s, which reflects the shift in the IC_50_ introduced by 4 Gy IR in the presence of the drug, as a measure of potential radiosensitizing activity. Cisplatin showed a low RER of 1.28–1.51 in all cell lines that were tested (Figure 2B). The RER of cetuximab was determined as 1.00 in UT-SCC-24a and 0.86 in UT-SCC-36, indicating lack of radiosensitization under our experimental conditions (Supplementary Figure 3B). Therefore, we did not investigate cetuximab any further in this manuscript.

Although olaparib showed a similar or somewhat higher RER (11.56) than GSK635416A (> 7.67) in UT-SCC-24a cells, GSK635416A acted as a considerably stronger radiosensitizer in the other two cell lines. Importantly, the reported IC_50_ (IRneg) values of ‘> 25 mM’ underestimates the radiosensitizing capabilities of GSK635416A since 25 µM is the highest concentration that was tested; as the actual IC_50_ is higher, a higher RER would be a consequence.

### GSK635416A sensitized a variety of cancer cell lines to radiation

To assess the breadth of impact of our new radiosensitizer, we also tested GSK635416A in two additional HNSCC cell lines (UT-SCC-2 and UT-SCC-8) and two tumor cell lines originating from other tissues (HeLa and A549) (Table 2). GSK635416A shows virtually no cytotoxicity in all tested cell lines (IC_50_ [IRneg] > 25 µM in UT-SCC-2, UT-SCC-8 and HeLa; 6.95 µM in A549), but effectively sensitized all cell lines to IR (RER 1.49 – 9.23). Once again, olaparib was found to be an efficient radiosensitizer (RER 2.90 – 13.46), while cisplatin only produced a limited radiosensitizing effect (RER 1.10 – 1.62). Of note, the RERs of cisplatin, olaparib and GSK635416A could not be directly compared to each other, as the RER for GSK635416A was underestimated given its limited cytotoxicity on non-radiated cells (the IC_50_ [IRneg] value of ‘> 25 µM’ underestimates the calculated ratio).

**Table 2:** The effect of GSK635416A on various cell lines, compared to cisplatin and olaparib

	UT-SCC-2			UT-SCC-8			HeLa			A549		
Inhibitor	IRneg	IRpos	RER	IRneg	IRpos	RER	IRneg	IRpos	RER	IRneg	IRpos	RER
GSK635416A	>25	4.32	**5.79**	>25	9.93	**2.52**	>25	2.71	**9.23**	6.95	4.68	**1.49**
Cisplatin	0.61	0.39	**1.56**	2.39	1.63	**1.47**	0.36	0.22	**1.62**	1.21	1.10	**1.10**
Olaparib	5.98	2.06	**2.90**	9.42	0.70	**13.46**	2.04	0.16	**12.75**	1.24	0.22	**5.64**

Calculated IC_50_ values (in mM) for IRneg and IRpos and the determined radiation enhancement ratio (RER) from dose-response curves of GSK635416A, cisplatin and olaparib in UT-SCC-2, UT-SCC-8, HeLa and A549 cells, as measured in a cell viability assay at day 7. (Data shown are the mean of three independent experiments, with SEM).

**Figure 3:**
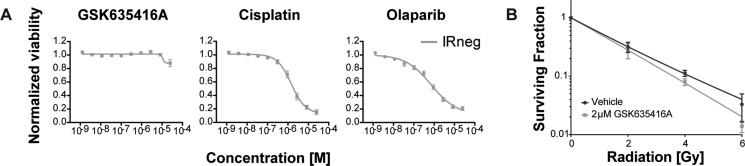
The effect of GSK635416A on normal BJ-ET fibroblast cells (**A**) Cytotoxicity of GSK635416A, cisplatin and olaparib treatment in a normal fibroblast cell line, BJ-ET. (Data shown are the mean of three independent experiments, with SEM.) (**B**) Clonogenic survival of BJ-ET cells cultured in the presence or absence of 2 μM GSK635416A and exposed to different doses of radiation. (Data shown are the mean of five independent experiments, with SD).

In keeping with the importance of selectivity for cancer cells during treatment, we subjected a normal fibroblast cell line (BJ-ET) to various radiosensitizing drugs (Figure 3A). Interestingly, GSK635416A showed significantly higher cell viability in these cells when compared to cisplatin or olaparib. Also, GSK635416A showed only modest radiosensitization (DEF_37_ 1.11, ± 0.16) at a concentration of 2 µM in these cells when measured in the CFA (Figure 3B). Additionally, PE was similar between vehicle and GSK6535416A treatments, implying no apparent cytotoxicity of this drug on these cells (Supplementary Figure 1B). Taken together, these data suggest that GSK635416A’s radiosensitization is tumor-specific in a variety of cancer cell lines with limited cytotoxicity in non-radiated cells and in non-transformed cells.

### GSK635416A targets ATM kinase

**Figure 4:**
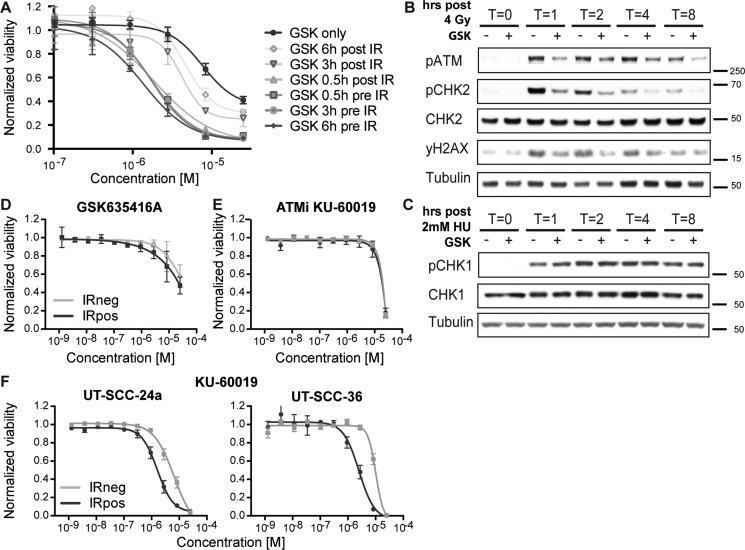
GSK635416A targets the DDR pathway (**A**) Tested timeframes of GSK635416A administration post- or pre-radiation in UT-SCC-36. (**B**, **C**) Western blot of UT-SCC-36, showing subunits of the DDR pathway. Cells were exposed to 4 Gy IR for ATM pathway activation (B), and with 2 mM Hydroxyurea for ATR pathway activation (C). Cells were treated in the presence (+) or absence (−) of 2 μM GSK635416A, and subsequently harvested 0, 1, 2, 4 or 8 hours following treatment. (**D**) GSK635416A in H23 ATM-deficient cells shows a loss of radiosensitization (1 Gy). (**E**) Lack of radiosensitization by the ATM inhibitor KU-60019 in H23, confirming ATM defect (1 Gy). (**F**) ATM inhibitor KU-60019 dose-response curves of UT-SCC-24a and UT-SCC-36 (4 Gy). (Data shown in A, D, E and F were measured with cell viability read-out at day 7 and shown as mean of at least three independent experiments with SEM).

Our screen identified a novel and unique radiosensitizer given its selectivity to cancer cells with limited cytotoxicity to non-radiated as well as normal cells. To identify the underlying biology and target for this compound, we first determined the timing of GSK635416A administration (i.e. prior to or following IR) that resulted in the most prominent radiosensitizing effect on HNSCC cells. GSK635416A exhibited a higher radiosensitizing effect when added prior to IR (0.5, 3 and 6 hours pre-IR; UT-SCC-36 Figure 4A, UT-SCC-24a Supplementary Figure 4A) than when added to cells post-IR, which suggested that GSK635416A targets the immediate DNA damage response (DDR). Given that the ataxia-telangiectasia mutated (ATM) kinase is an important early sensor of DNA double-strand breaks (DSBs) generated by IR, we examined IR-induced activation of the ATM pathway over time in the presence or absence of GSK635416A. We assayed phosphorylation of ATM and its key downstream target CHK2 as read-outs for activation of ATM signaling (UT-SCC-36 Figure 4B, UT-SCC-24a Supplementary Figure 4B). A marked decrease in phosphorylation of both ATM and CHK2 was observed in the presence of GSK635416A. Since phosphorylation of ATM is the result of autophosphorylation, this suggested that GSK635416A acts as a direct inhibitor of the ATM kinase. To test target specificity, we generated replication stress using Hydroxyurea (instead of DNA damage following IR) to activate ATM-related ataxia telangiectasia and Rad3 related (ATR) kinase (Figure 4C) and tested phosphorylation of its downstream target CHK1 in response to GSK635416A treatment. Notably, GSK635416A exhibited no effect on CHK1 phosphorylation, excluding the ATR kinase as a possible target of GSK635416A and further cementing specificity of this inhibitor for the ATM signaling pathway. Additionally, we tested 10 µM GSK635416A *in vitro* against a panel of 456 kinases (not including ATM) in a competition binding assay (Materials and Methods, Supplementary text), which did not reveal any additional targets (Supplementary Table 2). Due to its large molecular weight of around 350 kDa the associated challenges of expression and purification were difficult, therefore we chose to address whether ATM constitutes a valid target of GSK635416A by testing the radiosensitizing effect in the H23 cell line, that lacks ATM [19]. Of note, H23 cells were radiated with only 1 Gy instead of 4 Gy, because they are highly radiosensitive. The radiosensitizing activity of GSK635416A was lost in ATM deficient H23 cells upon 1 Gy of IR (Figure 4D). The lack of radiosensitization in two ATM deficient HNSCC cell lines (UPCI-SCC-040 and UPCI-SCC-131) [20] further supports ATM specificity of the radiosensitization by GSK635416A (Supplementary Figure 5). The established ATM-inhibitor KU-60019 also failed to radiosensitize H23 cells at 1 Gy, supporting the role of ATM deficiency of this cell line (Figure 4E), while exhibiting radiosensitizing activity in UT-SCC-24a and UT-SCC-36 cell lines at 4 Gy (Figure 4F). Notably, KU-60019, was not able to radiosensitize cells to the same extend as GSK635416A, and showed higher cytotoxicity (compare Figure 4F to Figure 2A; UT-SCC-24a and UT-SCC-36). Collectively, the above data indicate that IR-dependent cell kill incurred by GSK635416A requires ATM and suggests that the mechanism of GSK635416A action proceeds via inhibition of the DDR. We therefore assessed DSB formation by radiation with constant-field gel electrophoresis techniques and show increased DSBs after radiation when combined with GSK635416A. Together, this further supports GSK635416A’s role in DDR and as ATM inhibitor (Supplementary Figure 6).

### GSK635416A and olaparib interplay

**Figure 5:**
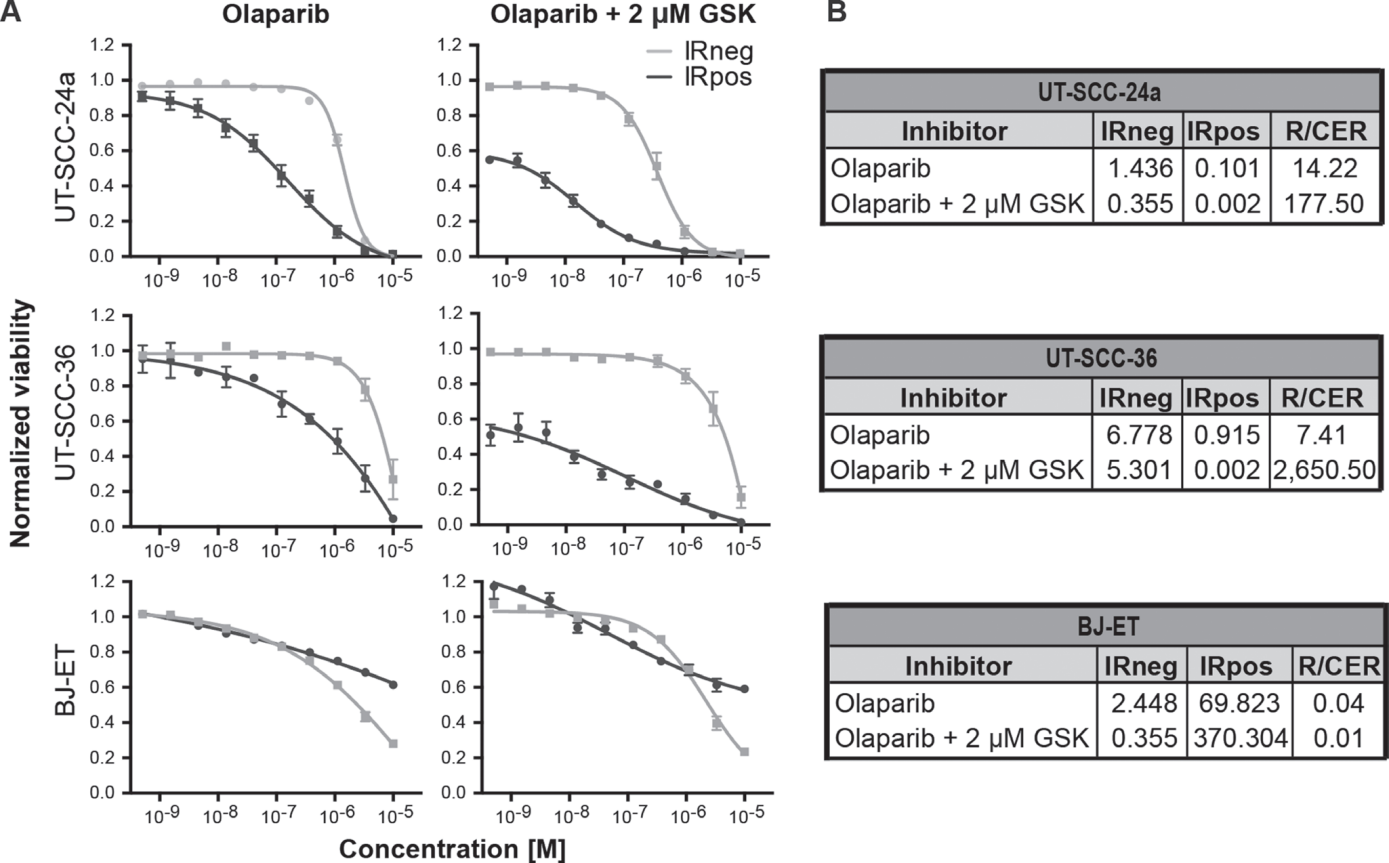
GSK635416A combined with olaparib enhances IR effect in radiosensitizing HNSCC cell lines but not in normal fibroblast BJ-ET cells (**A**) Dose-response curves of olaparib in the presence or absence of 2 μM GSK635416A in UT-SCC-24a, UT-SCC-36 and BJ-ET, measured by cell viability read-out at day 7. (**B**) Corresponding IC_50_ values (μM) for IRneg and IRpos, and the RER and CER were determined to compare the treatments. (Data are shown as mean of three to five independent experiments, with SEM).

While both olaparib and GSK635416A sensitize cells to radiation, they target different aspects of the DDR. While olaparib inhibits PARP, GSK635416A targets the ATM kinase. Here we tested whether combined inhibition of both pathways could improve radiosensitization without further increasing cytotoxicity of cells that are not exposed to IR. UT-SCC-24a and UT-SCC-36 were treated with or without 2 µM GSK635416A and with increasing olaparib concentrations up to 10 µM in combination with IR (Figure 5A). The RER for olaparib as a single drug is 14.22 and 7.41 in UT-SCC-24a and UT-SCC-36, respectively, while the combined enhancement ratio (CER) for olaparib and 2 µM GSK635416A increased 14- and 320-fold in the same cell lines (CER 177.50 and 2650.50 in UT-SCC-24a and UT-SCC-36, respectively; Figure 5B).

A different presentation of the data in Supplementary Figure 7A shows that olaparib radiosensitization is largely unaffected by GSK635416A addition. Simulating an additive effect by adding the effect of 2 µM GSK635416A (at the lowest olaparib concentration) to the non-GSK635416A treated olaparib viability values at different olaparib doses shows a “theoretical” curve line that is not different from the measured values when combining with GSK635416A. Comparing the IRneg profile of olaparib monotherapy to the IRneg curve of olaparib plus 2 µM GSK635416A revealed no increased cytotoxicity on non-radiated UT-SCC-36 cells (Figure 5A, IC_50_ [IRneg] 6.78 and 5.30, respectively) and increased cytotoxicity on non-radiated UT-SCC-24a cells (Figure 5A, IC_50_ [IRneg] 1.44 and 0.36, respectively). Most importantly and consistent with a lack of radiosensitization of GSK635416A in this cell line, the combination treatment did not show marked additional effects on cell viability of normal BJ-ET cells (Figure 5A) as ratio values remained low (RER 0.04 for olaparib and CER 0.01 for olaparib with GSK635416A) (Figure 5B).

The combination of GSK635416A with olaparib was also tested at 0.3 µM and 5 µM of GSK635416A (Supplementary Figure 7B and 7C), revealing a clear dose-dependent effect of GSK635416A in combination with olaparib with respect to radiosensitization. Although some cytotoxicity of GSK635416A was observed at 5 μM, as deduced from the IRneg curves starting at a cell viability below 1.0 (Supplementary Figure 7B, UT-SCC-24a and UT-SCC-36), no additional cytotoxicity of 0.3, 2 or 5 µM GSK635416A was observed for normal BJ-ET cells (Figure 5A and Supplementary Figure 7B) compared to olaparib alone. The CER in BJ-ET cells only increased when olaparib was combined with 5 µM GSK635416A (Supplementary Figure 7C, CER 0.58) due to the additional combination with IR. Collectively, these data suggest that GSK635416A increases radiation induced tumor cell death and maintains this property also in combination with olaparib, while preserving the low cytotoxicity profile in non-radiated and normal cells.

## DISCUSSION

In spite of various treatment attempts, advanced HNSCC is poised by poor prognoses, with RT constituting the best available therapy option next to surgery, despite its limited benefits when administered on its own. The addition of cisplatin or cetuximab to RT regimens has shown only limited survival benefit and substantial systemic toxicity compared to RT alone [6, 7, 9, 10]. While immunotherapy and newer drugs are under development for HNSSC, RT will remain an important part of the treatment protocol. Development of better and more specific radiosensitizers is crucial and may have substantial therapeutic effects on HNSCC patients. To identify novel radiosensitizers, we performed a screen with the GSK kinase inhibitor library to identify compounds capable of sensitizing HNSCC cells to IR, while excluding compounds targeting non-radiated cells. This approach was aimed at selecting compounds capable of improving current treatment efficacy and avoiding adverse effects. Using this approach, we identified one compound, GSK635416A, as a novel tumor-specific radiosensitizer.

GSK635416A, with a DEF_37_ of 1.99 in HNSCC cells, compares favourably to established radiosensitizers, including cisplatin (DEF_37_ of 1.90 in UT-SCC-24a [16]) and olaparib (DEF_37_ of 1.25 (± 0.18) and 1.61 (± 0.55) in 6 other UT-SCC cell lines at 1 µM and 3.3 µM, respectively [18]). Direct comparison to cetuximab was not assessed, as no DEF_37_ for this drug has been reported. In addition to its IR-dependent effects, an ideal radiosensitizer would be expected to display tumor-specific activity, resulting in limited systemic toxicities as well as sparing normal cells within the radiation field. Our data indicate that GSK635416A outperforms cisplatin, cetuximab and olaparib, as it did not affect viability of non-radiated HNSCC cells, was not cytotoxic to normal BJ-ET fibroblast and barely radiosensitized BJ-ET cells (DEF 1.11). High cytotoxicity of cisplatin, cetuximab and olaparib treatment was observed when these drugs were administered as single agents to a variety of cell lines, including BJ-ET cells. On the basis of these comparisons, GSK635416A has the potential for development into a highly effective and tumor-specific radiosensitizing compound applicable to difficult to treat head and neck cancers that often fail to respond to even high doses of radiotherapy.

Given the severe limitations of radiosensitizers currently administered in the clinic and the urgent need for new RT-compatible therapies, there has been substantial discussion on the topic. It has been proposed that inhibitors of the DDR pathway may present a suitable source of novel targeted anticancer treatments [21–26]. Interestingly, we found that GSK635416A appeared to inhibit DDR by targeting the ATM kinase. There are multiple arguments for this. First, GSK635416A must be present during, and not after, exposure to IR to act as a radiosensitizer, suggesting an effect on early cellular events resulting from IR. Second, because ATM acts upstream of the double-strand DNA repair pathway, inhibition of this master kinase in DDR could thus explain the strong radiosensitizing effects of GSK635416A. Indeed, exposure of HNSCC cells to GSK635416A markedly reduced activation of ATM and its downstream target CHK2 in response to IR. Thirdly, a cell line lacking ATM failed to be radiosensitized by GSK635416A. Furthermore, GSK635416A seems remarkably specific for ATM kinase as we failed to detect any other target for GSK635416A in an *in vitro* competition binding assay screen with 456 kinases. This may explain why GSK635416A hardly affects cells unless radiated, as the drug has few detectable off-targets. Of note, we compared the effects of GSK635416A to an established ATM inhibitor (KU-60019), which also displayed radiosensitizing activity, but was more toxic to non-radiated cells. It is possible that GSK635416A is simply more selective for ATM than other reported ATM inhibitors [24, 27], since GSK635416A has a distinct chemical structure. In literature, only a handful of selective ATM inhibitors have been reported, all in the interest of finding novel radiosensitizers. These ATM inhibitors have not been tested on HNSCC cell lines and did not progress into the clinical practice due to their poor bioavailability and selectivity [24, 27].

Deciphering the molecular targets of bioactive molecules is a key step towards understanding their clinical potential, particularly in designing effective combination therapies while mitigating compounding side effects. As ATM is critical in DNA double strand break repair, attenuating this repair by inhibiting ATM could simply explain the molecular basis for GSK635416A as a radiosensitizer. As an inhibitor of ATM, GSK635416A affects the DDR pathway. Simplified, the DDR pathway is activated by single (SSB) and double-strand DNA breaks (DSB). SSBs are recognized mainly by PARP [28], and ATM is activated by DSBs [29]. Olaparib inhibits PARP and thus plays an important role in the base-excision repair (BER) pathway and in the repair of SSBs. The radiosensitizing effect of olaparib requires DNA replication which implies selectivity of rapidly dividing and/or DNA repair defective tumor cells. Bryant et al. showed that PARP inhibitors selectively kill homologous recombinant (HR)-deficient (BRCA2) cancers cells [30]. In addition, Verhagen et al. and Wurster et al. showed that olaparib has stronger synergistic interaction in HR-deficient than in HR-proficient HNSCC once combined with IR [18, 31]. Unfortunately, in HNSCC mutations in HR genes are rare [31]. However, by inhibiting ATM, GSK635416A also inhibits HR. The accumulation of SSBs in the absence of PARP activity, leads to replication fork collapse and DSBs, which require HR factors to repair. IR produces DNA damage and SSBs that the replication fork encounters but perhaps may have controlled if the DDR would not have been inhibited by GSK635416A. This provides a rationale to explore the combined effect of PARP and ATM inhibitors as radiosensitizers. We show that the radiosensitizing effect of the combination of 2 µM GSK635416A and olaparib follows an additive effect. This effect could be further investigated in the future by varying concentrations of GSK635416A and by performing colony forming assays and *in vivo* experiments. Importantly, GSK635416A differs from olaparib in that it is considerably less cytotoxic in the absence of IR and less cytotoxic in healthy normal fibroblasts both in the presence or absence of additional IR. Therefore, we still believe that GSK635416A is an excellent lead for further development towards a radiosensitizing drug, either as single compound or in combination with olaparib, being a starting point for medicinal chemistry on its chemical structure with a corresponding target and biological mechanism. Furthermore, GSK635416A displayed radiosensitizing effects in cervical HeLa and lung A549 cancer cells, implying therapeutic potential against other cancer types. We expect that additional medicinal chemistry efforts to optimize GSK635416A, or other ATM inhibitors, may fuel a much needed improvement in treatment options for HNSCC patients, as well as other cancer patients that respond poorly to standard chemoradiotherapy.

## MATERIALS AND METHODS

### Cell culture

The human HNSCC cell lines UT-SCC-2, UT-SCC-8, UT-SCC-24a, UT-SCC-36 and UT-SCC-40 were kindly provided by Prof. R. Grénman (University of Turku, Finland). We primarily selected p53 mutated and HPV negative cell lines since 74% of HNSCC tumors are HPV negative and have poor prognosis [32]. Of these, the majority (75 – 85%) have TP53 mutations [33]. Cell lines with these characteristics were therefore chosen. These cell lines were harvested from previously untreated HPV negative patients and have various sensitivities to IR [34, 35]. Cell line characteristics are listed in Table 1. These cells were cultured in Dulbecco’s Modified Eagle Medium high glucose, GlutaMAX™, pyruvate (Invitrogen) supplemented with 10% FBS, 1% non-essential amino acids (Sigma), penicillin and streptomycin (Gibco 15070, 50 Units/ml and 50 μg/ml), as previously described [36, 37]. The characterisation of these cell lines was further confirmed by immunohistochemistry staining of hematoxylin-eosin, Cytokeratin AE1/3, Cam 5.2, p63 and Vimentin. Two human lung cancer cell lines (A549 and H23 [ATCC CRL-5800]), a human cervical cancer cell line (HeLa), and a human normal fibroblast cell line (BJ-ET [ATCC CRL-2522, overexpressing hTERT] [38]) were cultured in DMEM (Invitrogen) supplemented with 10% FBS and penicillin and streptomycin. The cell lines were cultured at 37°C with 5% CO_2_.

**Table 1:** HNSCC cell line characteristics

Cell line	Gender	Primary tumor location	TNM	Type of lesion	Histol. grade	Radiosens. (SF2 ± SD)	HPV	P53	Ref
UT-SCC-2	Male	Floor of mouth	T4N1M0	Primary	2	0.35 ± 0.05	Neg	Mut	[34, 35, 37]
UT-SCC-8	Male	Supraglottic larynx	T2N0M0	Primary	1	0.37 ± 0.03	Neg	Mut	[34, 37]
UT-SCC-24a	Male	Tongue	T2N0M0	Primary	2	0.51 ± 0.06	Neg	Mut	[35, 37]
UT-SCC-36	Male	Floor of mouth	T4N1M0	Primary	3	0.72 ± 0.07^*^	Neg	Mut	[37]
UT-SCC-40	Male	Tongue	T3N0M0	Primary	1	0.45 ± 0.02^†^	Neg	ND	[37]

TNM status of primary tumors according to the International Union against Cancer (1997).

Histologic grade: 1, well differentiated; 2, moderately differentiated; 3, poorly differentiated.

Radiosens.: radiosensitivity. ^*^Determined in this manuscript. ^†^Unpublished data from Prof. R. Grénman.

SF_2_: Survival fraction at 2 Gy, measured by clonogenic survival.

HPV Neg: human papillomavirus negative.

P53 Mut: mutated, ND: not detectable.

### Compounds

We exposed the cell lines to the open-source GlaxoSmithKline Published Kinase Inhibitor Set (GSK PKIS) containing 356 defined and potential protein kinase inhibitors, representing 31 chemical chemotypes [39]. The majority of kinase inhibitors in this screening library compete with ATP for binding to the common enzyme active site.

The following individual compounds were used. Olaparib was obtained from Syncom (Groningen, The Netherlands). Cisplatin and ATM-inhibitor KU-60019 were obtained from Selleck Chemicals (Houston, USA). Cetuximab (Erbitux, 5 mg/ml, buffer) was obtained from Merck Serono (Darmstadt, Germany). GSK635416A was synthesized as described [40], and stock solution was dissolved in 20% DMSO and 80% Ethanol at 10 mM. Compounds dissolved in solely DMSO were added automatically to the plates with the HP D300 Digital Dispenser. Hydroxyurea (HU, a ribonucleotide reductase inhibitor) was obtained from Sigma.

### Screening

Using a robotic liquid handling platform system, we screened the compound library in three ten-fold dilutions (50 nM to 5 µM) in three cell lines (UT-SCC-24a, UT-SCC-36 and UT-SCC-40) with or without 4 Gy IR). All experiments were performed in independent biological triplicates. On day 0, cells were seeded automatically (Thermo Scientific Multidrop Combi Reagent Dispenser) in 384-well plates in 45 µl medium. Seeding densities were previously optimized to reach approximately 80% confluency on day 7. The outer two rows and columns of the 384-well plates did not include any experimental or control compounds to exclude potential evaporation and edge effects. At day 1, compounds were administered with the ‘Hamilton STARlet Liquid Handler’ robot, and DMSO and phenylarsine oxide (PAO, 20 µM) were used as a negative and positive control for cell viability, respectively. Furthermore, olaparib was taken along as a control for detecting radiosensitizing effects [18]. Half an hour after compound addition, the plates were either subjected to 4 Gy IR (Best Theratronics Gammacell^®^ 40 Exactor, 0.95 Gy/min, Ottawa, Ontario, Canada) (IRpos) or left non-radiated (IRneg). At day 7, cell viability was determined by CellTiter-Blue assay. In short, cells were incubated with CellTiter-Blue® (Promega, final 1:20) for 4 hours, then the fluorescence intensity was measured using the EnVision plate reader (Perkin Elmer).

### Hit validation

Lead candidates showing the largest mean difference with significant adjusted *p*-values, were selected for validation. We picked the best 5 compounds from the following four categories: dataset at 50 nM, 500 nM, 5 µM and all concentrations combined. The efficacy of the 17 selected compounds was validated using freshly dissolved compounds. This was done in 3-fold dilutions with 10 concentrations ranging from 2 nM to 40 µM on UT-SCC-24a, UT-SCC-36 and UT-SCC-40 cell lines, in triplicate in 384-well plates.

Thereafter, we selected the top hit, GSK635416A, based on the largest window between IRneg and IRpos. We resynthesized GSK635416A [40] to chemically validate for purity by High-performance liquid chromatography and for structure by mass spectrometry. Subsequently, we biologically validated its activity on our panel of cell lines (UT-SCC-2, -8, -24a, -36 and -40, HeLa, A549, and BJ-ET) in 3-fold dilutions with 10 concentrations ranging from 1.3 nM to 40 µM. All subsequent validation experiments were performed with the resynthesized GSK635416A in 96-well format routinely.

### Colony formation assay

To validate the efficacy of our lead candidate, we assessed clonogenic survival after radiation using the colony formation assay, as described [41]. Briefly, single-cell suspensions of proliferating UT-SCC-36 and BJ-ET cells were seeded into 10-cm dishes at different cell densities in triplicate and radiated 6 hours after plating. Cells were exposed to a single radiation dose, varying from 2 to 6 Gy. GSK635416A was added 1 hour prior to IR at 2 µM. Controls were treated with the vehicle (drug solvent, DMSO/ethanol) at equal concentration as the GSK635416A treated cells. After 2 weeks (for UT-SCC-36) or 3 weeks (for BJ-ET) of incubation, colonies were fixed and stained with 0.5% crystal violet/6.0% glutaraldehyde. Only colonies consisting of more than 100 cells were counted. GSK635416A treated samples did not require longer incubation times as GSK635416A did not influence colony formation or size at this concentration. Plating efficiencies were not significantly altered by GSK635416A treatment (Supplementary Figure 1). Survival after radiation of vehicle or GSK635416A treated cells was calculated relative to the plating efficiency of non-radiated controls, vehicle or GSK635416A treated cells, respectively. Survival data points are the mean of the averages of three to five independent experiments. Dose enhancement factors (DEF) values were calculated as the ratio of radiation doses to produce 37% survival (DEF_37_) without GSK635416A to those with GSK635416A. These doses were calculated from the linear quadratic fits through the radiation dose response data.

### Western blot analysis

Western blot analysis was performed using standard protocols, to determine the target of GSK635416A. In brief, UT-SCC-24a and UT-SCC-36 cells were lysed directly with Laemmli sample buffer. Samples were separated by SDS-PAGE and proteins transferred to PVDF membranes (Millipore). The PVDF membranes were subsequently blocked by 5% milk in TBS. Antibody blotting was done in TBS supplemented with 0.05% Tween and 2% milk. Antibodies used for Western blotting: pCHK1-Ser345 (Cell Signaling; 133D3), CHK1 (Santa Cruz Biotechnology; G-4), pCHK2-Thr68 (Cell Signaling), CHK2 (Santa Cruz; H-300), pATM-S1981 (Rockland Immunochemicals for research), H2AX-Ser139P (Upstate) and Tubulin-α (Sigma).

### Data analysis and radiosensitization

Analysis of the screening data was done using R version 3.1.2. Cell viability data were analysed using the normalized percent inhibition (NPI) method, to correct for plate effects and allow direct comparison of plates [42]. This NPI method divides the difference between the average of the positive controls and the compound measurement, by the difference between the averages of the positive and negative controls. This way, the value ‘0’ corresponds to complete cell death and the value ‘1’ to no treatment. Correlation plots of the replicates showed consistent correlation between the three replicates. The effect of IR was eliminated by normalizing to negative controls that received IR, which allowed us to evaluate the enhanced effects of compounds with IR. If a compound showed identical viability in the absence and presence of IR, there would be no enhanced effect. If a compound in the IRpos group showed decreased viability compared to compound alone at the same concentration, potential radiosensitizing effect would be identified. Therefore, potential radiosensitization was determined by the difference between IRpos and IRneg NPI values gathered for each compound, for all tested conditions (three cell lines, three concentrations and three replicates). We then compared the distribution of the difference values of a compound to the distribution of the difference values of the negative controls. The comparison was done using the Wilcoxon test. The resulting *p*-value was corrected for multiple testing using the Benjamini-Hochberg method. Adjusted *p*-values ≤ 0.1 were considered significant.

All other analyses, such as compound potency determination, were performed using Graphpad Prism version 6.0h. Normalized data were fitted using nonlinear regression dose-response curves. To calculate the absolute IC_50_ from the fitted curve we determined the interpolation of Y = 0.5 with the corresponding X-value of the curve. We determined ratios to define the enhanced effect of combined treatments. The radiation enhancement ratio (RER) was defined as: IC_50_ (drug alone) / IC_50_ (drug + 4 Gy IR); with a RER value of > 1 being indicative for radiosensitization. The combined enhancement ratio (CER) was defined as: IC_50_ (olaparib + 2 µM GSK635416A) / IC_50_ (olaparib + 2 µM GSK635416A + 4 Gy IR).

## SUPPLEMENTARY MATERIALS FIGURES AND TABLES




