# Supplementary material for: Identification of a novel ATM inhibitor with cancer cell specific radiosensitization activity
Source: Oncotarget. 2017 May 19;8(43):73925–37. doi: 10.18632/oncotarget.18034 (PMC5650312; doi:10.18632/oncotarget.18034)
Supplement: Supplementary file 1 [file oncotarget-08-73925-s001.pdf]

Identification of a novel ATM inhibitor with cancer cell specific radiosensitization activity

Supplementary Materials

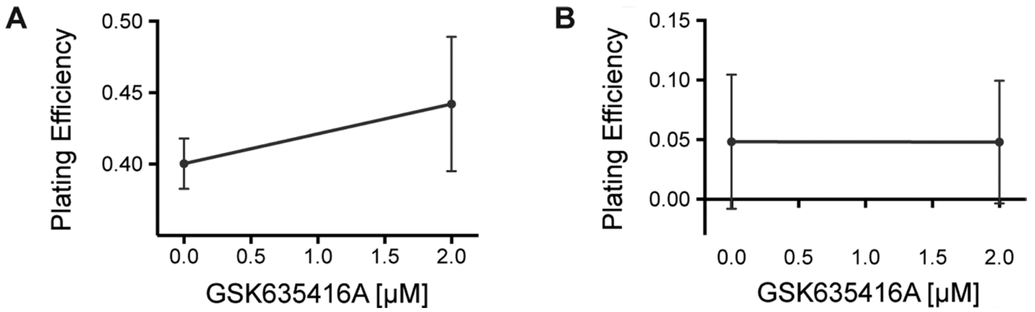

Supplementary Figure 1: GSK635416A cytotoxicity as determined with a colony forming assay. Plating efficiency did not decrease by GSK635416A. Plating efficiency of UT-SCC-36 (**A**) at 2 μM GSK635416A compared to BJ-ET (**B**) These results suggest that 2 μM GSK635416A does not affect clonogenic survival. (Data are shown as mean of three (UT-SCC-36) and five (BJ-ET) experiments, with SD.)

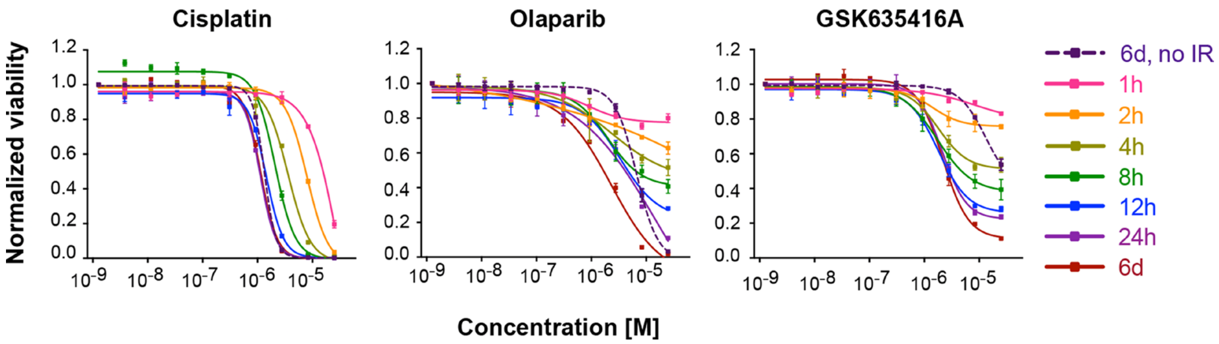

Supplementary Figure 2: Drug wash-out experiment in UT-SCC-36. At Day 1 UT-SCC-36 cells were treated for half an hour with cisplatin, olaparib or GSK635416A and subsequently irradiated with 4 Gy. At the indicated time points post irradiation, the drugs were removed by washing twice with medium. Data from cells treated with drug, but without IR, are plotted with a dash line. (Data are cell viability read-outs at day 7 and shown as mean of two independent experiments with SD).

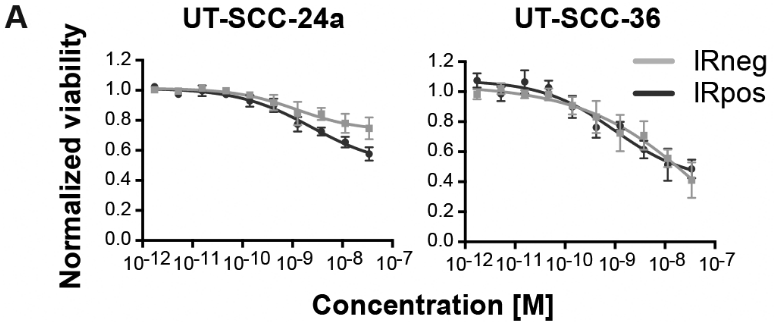

B

Cetuximab			
**Inhibitor**	**IRneg**	**IRpos**	**RER**
UT-SCC-24a	> 34	>34	1.00
UT-SCC-36	0.018	0.021	0.86

Supplementary Figure 3: Radiosensitizing properties of cetuximab. (**A**) Dose-response curves of cetuximab in UT-SCC-24a and UT-SCC-36, measured with cell viability read-out at day 7. (**B**) IC50 values (nM) for IRneg and IRpos were calculated from the corresponding dose-response curves and the RER was determined. (Data are shown as mean of three independent experiments, with SEM).

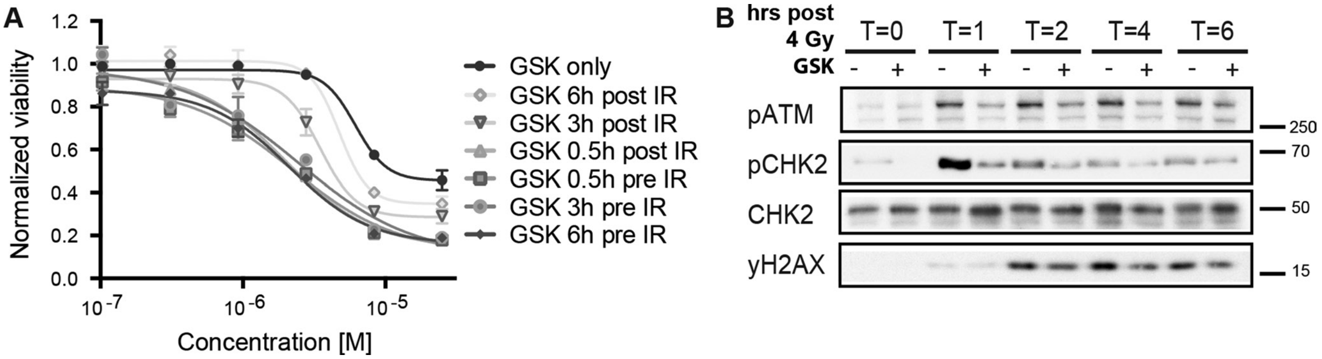

Supplementary Figure 4: GSK635416A targets the DNA damage response pathway. (**A**) Tested timeframes of GSK635416A administration pre- and post-radiation in UT-SCC-24a. (Data shown are measured with cell viability read-out at day 7 and presented as mean of at least three independent experiments, with SEM.) (**B**) Western blot of UT-SCC-24, showing various proteins involved in the DDR pathway. Cells were treated with 4 Gy IR in the presence (+) or absence (−) of 2 μM GSK635416A, and subsequently harvested 0, 1, 2, 4 or 6 hours following treatment. Western blots are probed with antibodies detecting the proteins or phosphoproteins indicated. The position of molecular weight standards is indicated on the right side. A marked inhibition of the ATM pathway components is observed.

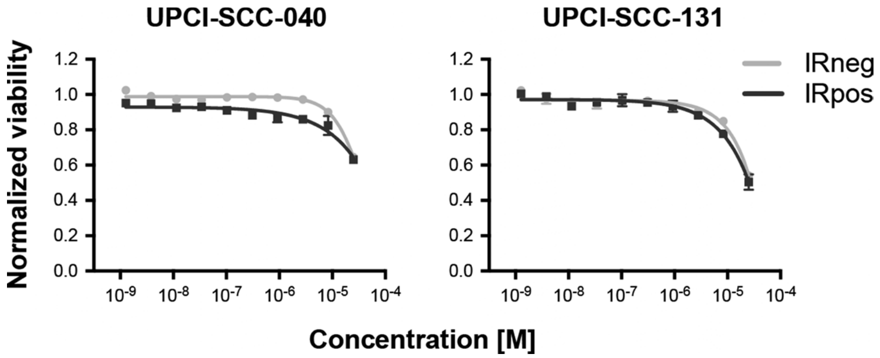

Supplementary Figure 5: Loss of GSK635416A-mediated radiosensitization in ATM deficient HNSCC cell lines, namely UPCI-SCC-040 and UPCI-SCC-131 as indicated. Cells were radiated with 2.5 Gy, IRpos, and compared to non-radiated plates, IRneg. (Data shown are collected form cell viability read-outs at day 7 and shown as mean of two independent experiments with SD).

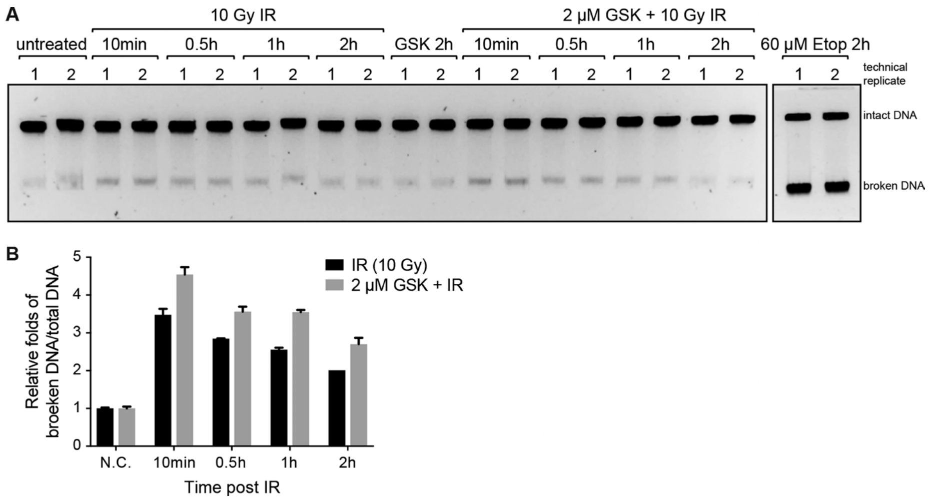

Supplementary Figure 6: Constant-field gel electrophoresis data show increased radiation induced DNA damage in the presence of GSK635416A. (**A**) Constant-field gel electrophoresis in UT-SCC-36 cells, collected at the indicated time points post 10 Gy IR in the presence and absence of 2 μM GSK635416A. Lower bands represent the broken DNA and the top bands the intact DNA. (**B**) Quantification of the broken DNA relative to intact DNA after scanning the gel, followed by calculation with ImageJ. (Data shown is a representative figure of two independent experiments).

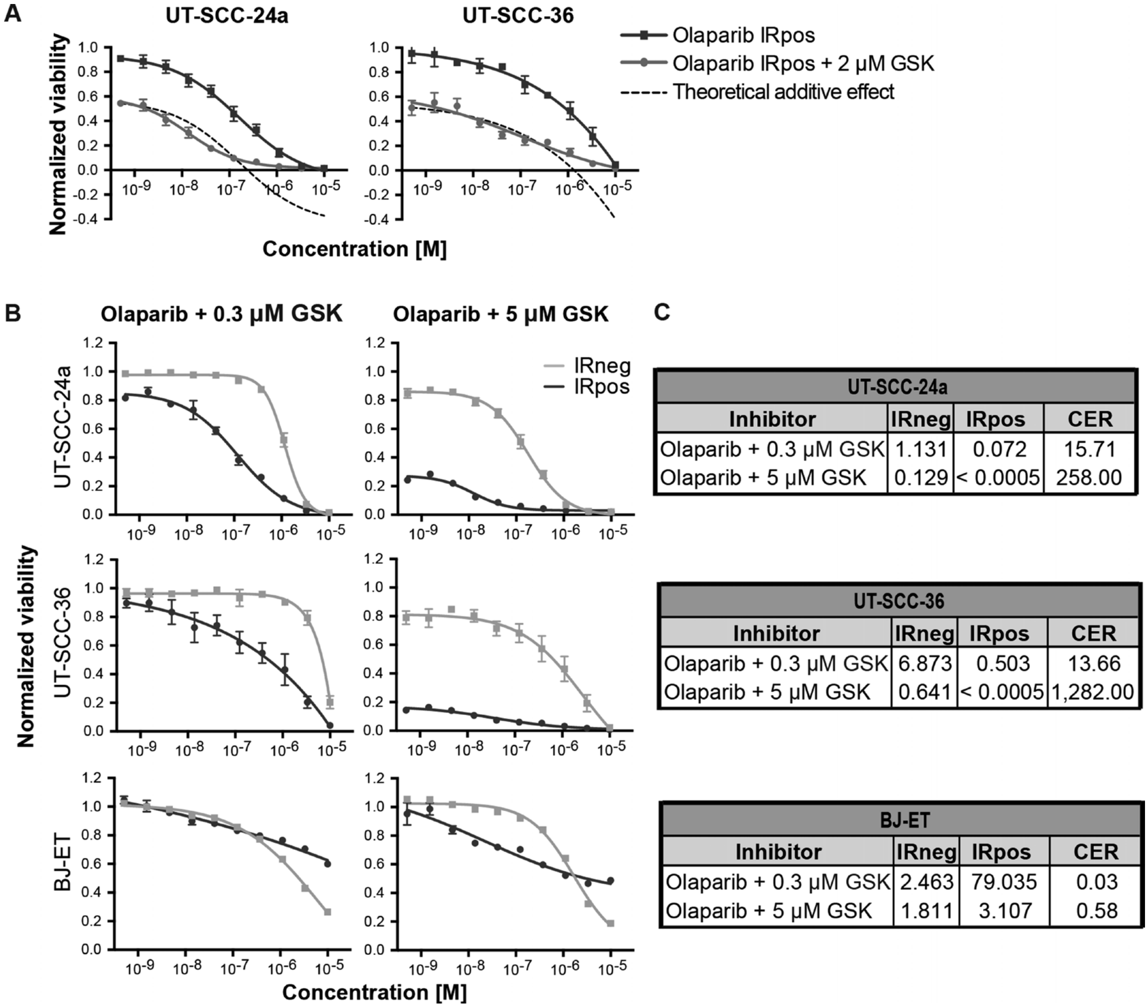

C

UT-SCC-24a			
Inhibitor	IRneg	IRpos	CER
Olaparib + 0.3 µM GSK	1.131	0.072	15.71
Olaparib + 5 µM GSK	0.129	< 0.0005	258.00

UT-SCC-36			
Inhibitor	IRneg	IRpos	CER
Olaparib + 0.3 µM GSK	6.873	0.503	13.66
Olaparib + 5 µM GSK	0.641	< 0.0005	1,282.00

BJ-ET			
Inhibitor	IRneg	IRpos	CER
Olaparib + 0.3 µM GSK	2.463	79.035	0.03
Olaparib + 5 µM GSK	1.811	3.107	0.58

Supplementary Figure 7: Radiosensitization by combining GSK635416A with olaparib. (**A**) This figure is related to the dose response curves in Figure 5 and includes an additional theoretical line that plots the calculated (in contrast to the tested) effect of 2 µM GSK635416A in combination with olaparib and IR assuming additivity. The same IRpos of olaparib and olaparib + 2 µM GSK635416A from Figure 5 is plotted. The dotted theoretical line depicts the additional effect of 2 µM GSK635416A at data point 0.5 nM olaparib (lowest concentration). This effect was subtracted from every data point of the 'Olaparib IRpos' curve, creating the parallel dotted theoretical line. This calculated line is similar to the 'Olaparib IRpos + 2 µM GSK635416A' curve, suggesting an additive effect. (**B**) Shown on the left are the dose-response curves of olaparib in the constant presence of 0.3 or 5 µM GSK635416A in UT-SCC-24a, UT-SCC-36 and BJ-ET. Depicted on the right (**C**) are the corresponding calculated IC50 values (µM) for IRneg and IRpos and the determined RER.. The figure shows that GSK635416A should be included at proper concentrations as 0.3 µM GSK635416A did not show an additive effect when compared to olaparib alone (see Figure 5). The combination of 5 µM GSK635416A with olaparib has increased radiosensitization effects when compared to olaparib as a single drug in Figure 5. (Data are shown as mean of three to five independent experiments, with SEM).

MATERIALS AND METHODS

Cell culture

UPCI-SCC-040 and UPCI-SCC-131 (DSMZ, Germany) were cultured in MEM supplemented with FBS, L-glutamine and non-essential amino acids, at 37°C under 5% CO_2.

Competition binding assay

GSK635416A was send to DiscoveRx UK for MAX KINOMEscan analysis. This scan included 456 human kinases and disease relevant mutant variants. GSK635416A was screened at 10 µM. A percentage of control ([test compound signal – positive control signal]/[negative control signal – positive control signal] *100) was determined for every kinase. Lower %Ctrl were associated with tighter binding. A Selectivity Score, excluding mutant variants, was determined as a quantitative measure of compound selectivity. This was calculated by dividing the number of kinases with affinity for the compound, by the total number of distinct kinases tested.

Constant-field gel electrophoresis

To directly detect DNA double-strand breaks induced by IR or by the combination of IR and GSK635416A, DNA from UT-SCC-36 cells was analysed by Constant-field gel electrophoresis [1] was performed on UT-SCC-36 cells. Cells were collected at various indicated time points post 10 Gy IR in the presence and absence of 2 µM GSK635416A. Cells exposed to 2 µM GSK635416A were first treated for 0.5 hour and consequently irradiated with 10 Gy. 2 h cell exposure to 60 µM Etoposide served as a positive control for drug induced DNA breaks. Quantification of the broken DNA was done with ImageJ. The percentage of broken DNA was normalized to untreated (N.C., negative control) samples.

REFERENCES

1. Wlodek D, Banath J, Olive PL. Comparison between pulsed-field and constant-field gel electrophoresis for measurement of DNA double-strand breaks in irradiated Chinese hamster ovary cells. Int J Radiat Biol. 1991; 60:779–90.

Supplementary Table 1: Results from validation of the 17 leading candidates

Compound	all.IRneg.Mean	all.IRpos.Mean	all.diff.Mean	all.w.pval	all.w.pval.adj
**GSK635416A**	0.917	0.720	**−0.197**	0.000000	0.000000
**SB-698596-AC**	0.654	0.554	**−0.100**	0.000030	0.000228
**GSK619487A**	0.654	0.573	**−0.081**	0.000008	0.000075
**GW578748X**	0.869	0.791	**−0.078**	0.010300	0.025800
**GW781673X**	0.722	0.647	**−0.076**	0.004190	0.014100
**GW683134A**	0.735	0.664	**−0.071**	0.003820	0.014100
**SB-678557-A**	0.774	0.706	**−0.068**	0.004930	0.014100
**SB-675259-M**	0.534	0.467	**−0.066**	0.005170	0.014100
**GSK269962B**	0.737	0.675	**−0.062**	0.000987	0.005920
**GSK238063A**	0.768	0.707	**−0.060**	0.001860	0.009280
**GW620972X**	0.636	0.597	**−0.039**	0.252000	0.359000
**GW810576X**	0.484	0.446	**−0.039**	0.341000	0.439000
**GSK1007102B**	0.381	0.344	**−0.038**	0.041600	0.096000
**GSK238583A**	0.725	0.692	**−0.034**	0.071800	0.127000
**GW806290X**	0.396	0.383	**−0.013**	0.230000	0.345000
**GW771127A**	0.816	0.809	**−0.007**	0.949000	0.949000
**GSK571989A**	0.307	0.341	**0.034**	0.802000	0.830000

GSK635416A significantly shows the largest mean difference between IRpos and IRneg for all variables (cell line, concentration, replicate) included.
all.IRneg.Mean: the mean of normalized values of non-radiated compounds for all variables.
all.IRpos.Mean: the mean of normalized values of radiated compounds for all variables.
all.diff.Mean: the mean of all single difference values. Difference values were determined by subtracting IRpos from IRneg for every single variation.
all.w.pval: compare the distribution of all single difference values to the distribution of the difference values of the negative controls using the Wilcoxon test, and calculate a *p*-value.
all.w.pval.adj: the resulting *p*-value was corrected for multiple testing using the Benjamin-Hochberg method. Adjusted *p*-values ≤ 0.1 were considered significant.

Supplementary Table 2: Results from competition binding assay with GSK635416A

Supplementary Table 2A: See Supplementary_Table_2A.

Supplementary Table 2B

Compound	Selectivity Score Type	Number of Hits	Number of Non-Mutant kinases	Screening Concentration (nM)	Selectivity Score
GSK635416A	S(35) *	0	395	10000	0
GSK635416A	S(10) *	0	395	10000	0
GSK635416A	S(1) *	0	395	10000	0

Competition binding assay to quantitatively measure interactions between GSK635416A and 456 human kinases and disease relevant mutant. A, Our %Ctrl results revealed no hits (average 90, range 56 – 100. Supplementary Table 2A). B, Selectivity Score results showed no hits as well.
*S(35) = (number of non-mutant kinases with %Ctrl < 35)/(number of non-mutant kinases tested)
*S(10) = (number of non-mutant kinases with %Ctrl < 10)/(number of non-mutant kinases tested)
*S(1) = (number of non-mutant kinases with %Ctrl < 1)/(number of non-mutant kinases tested)
